# Predicting cancer-related mycobiome aspects in gastrointestinal cancers: a systematic review

**DOI:** 10.3389/fmed.2024.1488377

**Published:** 2024-11-29

**Authors:** György Szklenarik, Peter Kiraly, Gabor Szegvari, David Dora, Zoltan Lohinai

**Affiliations:** ^1^Translational Medicine Institute, Semmelweis University, Budapest, Hungary; ^2^Department of Anatomy, Histology and Embryology, Semmelweis University, Budapest, Hungary

**Keywords:** mycobiome, gastrointestinal cancer, Ascomycota, Basidiomycota, case–control study, Shannon diversity index

## Abstract

**Background:**

Colonization of the human gut and tumor tissue by non-pathogenic fungi has emerged as a potential risk factor associated with cancer epidemics. Therefore, we aimed to conduct a systematic review to assess the role of fungal colonization in gastrointestinal (GI) tumors in increasing diagnostic efficiency.

**Methods:**

A PubMed citation search was conducted for publications up to and including March 2023, followed by full-text screening. Results were reported according to Preferred Reporting Items for Systematic Reviews and Meta-Analyses (PRISMA) 2020 guidelines. According to the Patient, Intervention, Comparison, Outcome (PICO) framework, patients diagnosed with early-and advanced-stage GI cancers, GI adenoma patients, and healthy subjects were included with metagenomic (MG) or internal transcribed spacer (ITS) sequencing on tumor tissue, adjacent normal tissue, stool, and blood samples.

**Results:**

Fourteen studies were eligible based on the inclusion criteria and methodological quality. Studies were conducted in stool (*n* = 8) or tissue (*n* = 7) as the most common specimens to be used for molecular analysis. In the collected data, ITS was used in *n* = 10 cases and metagenomic analyses in *n* = 3 cases. Observing the interindividual variability, we found that the Ascomycota/Basidiomycota (A/B) ratio from healthy to cancer state decreased in *n* = 2, increased in *n* = 1 cases, and did not change significantly in *n* = 2 studies. An increase in the relative abundance of Malassezia was identified in *n* = 4, of Candida in *n* = 5, of Saccharomyces in *n* = 2, and of Aspergillus in *n* = 2 cases. Intraindividual differences in the A/B ratio were identified in cancer and adjacent tissue (*n* = 4) and cancer vs. stool (*n* = 1) studies. Intraindividual variability of the A/B ratio showed an increase in *n* = 2 and no change in *n* = 3 studies for cancer tissue.

**Conclusion:**

In conclusion, the advent of highly sensitive sequencing methods may aid in the identification and the differentiation of cancerous from healthy human fungal colonizations with potential future diagnostic applications. Further studies are needed to establish reliable biomarkers for GI cancer screening.

## Introduction

The current literature on the microbiome aspects of gastrointestinal (GI) cancers mainly focuses on bacteria and viruses. However, fungi function by shaping innate and adaptive immunity and present a complex relationship with the host, impacting the homeostasis between the host and commensals (1). There has always been a strong interest in distinguishing adenoma or non-cancer tissue from cancer in different samples. Advances in mycobiome ecology are expected to provide future screening options or novel targets for future prevention and new directions in pathogenesis research. With an estimated 4.8 million new cases and 3.4 million deaths worldwide in 2018, GI cancers account for more than a quarter (26%) of global cancer incidence and more than a third of all cancer deaths (2). Primary malignancies of the GI tract, namely gastric cancer (GC), hepatocellular carcinoma (HCC), pancreatic adenocarcinoma (PADC), and colorectal carcinoma (CRC) share some common risk factors but differ primarily in their etiology and descriptive epidemiological profile (2, 3). Of the 5.1 million different species of fungi worldwide, approximately 300 regularly cause invasive disease in humans (4). The fungal community, also known as the mycobiome, represents less than 1% of the human gut microbiota, and they are involved in disease pathogenesis (5). The unique feature of the healthy gastrointestinal tract fungal ecology is that it begins in a more oxygen-rich and acidic environment (stomach) and continues in an increasingly oxygen-deprived environment (6). Thus, the stomach mycobiome had the highest average diversity, followed by the colon. Individual differences in the mycobiome remained relatively similar along the GI tract based on dispersion estimates. However, there was no reduction in dispersion in the lower GI or feces, and individual variation remained similar to the upper GI bacteriome (6).

Attention is being directed to the commensal fungi of the GI tract. The most commonly found fungal genera inhabiting the physiological GI system are Candida, Saccharomyces, and Cladosporium (Dikarya Subkingdom, Ascomycota et Basidiomycota Phylums) (7). In the 2017 Human Microbiome Project, the fungal communities were characterized by the high prevalence of Saccharomyces, Malassezia, and Candida, along with *S. cerevisiae*, *M. Restricta*, and *C. albicans*. In practical terms, it is found that the Ascomycota and Basidiomycota phyla are almost only present in the human gut, while other phyla are only found in isolated fungal infections or poisoning or allergic processes.

These eukaryotic cells can assume a variety of morphologies, express antigens that are distinct from bacteria (8), and can show an association with certain cytokines and interleukins (9, 10). The mycobiome is an important part of the activation of innate, type 17, and B-cell-mediated immunity in gut health and pathogenic processes (11), and the result of colonization with intestinal fungal species (including *C. albicans*) is fundamentally unique from commensal bacteria. Others showed associations of gut fungal microbiota and central nervous system (CNS) diseases (12) (gut–brain axis) including multiple sclerosis (13), Alzheimer’s (14, 15) Parkinson’s (16), schizophrenia (17), and lung diseases (gut–lung axis) (18), such as bronchiectasis (19), asthma bronchiale (20), lung cancer (21) or inflammatory bowel diseases (IBDs) (22), and irritable bowel syndrome (IBS) (23–26).

Advanced technologies in the area of sequencing have provided comprehensive tools to detect not only bacteria but also fungal components of the gut microbiome (27). The emerging understanding of fungal species in the gut ecology revealed new insights into health and disease (28). Recent data suggest that tumor tissue also contains bacterial and fungal elements using FISH (28S rRNA sequences) (29), IHC staining for anti-b-glucan, and fungal cell wall-specific Gomori methenamine silver, both with high false negative and positive rates. The intratumoral DNA is now widely studied using high-throughput sequencing to identify oncology biomarkers, which holds the potential to identify yeast components with potential relationships to the tumor microenvironment (30). Additionally, others showed that adjacent normal tissue from cancer patients also possesses microbiota with lower abundance than tumor tissues (11). The presence of fungal components was found intratumorally in pancreatic, breast, and ovarian cancer (29, 31). Others showed that macrophages in CRC, and other cancers, including melanoma, lung cancer, glioma, breast cancer, and HCC show diverse spatial distribution (32). Bacterial dysbiosis causes activation of macrophages and translocation of bacteria, leading to the development of chronic pro-tumorigenic inflammation (33). The weakening of the macrophage barrier decreases the tumor-killing function of CD8+ T cells by reducing their activation and infiltration (34).

Studies showed cancer-type-specific fungal ecologies in tumor tissue of melanoma, breast, pancreatic, ovary, lung glioblastoma, bone, and pan-cancer cohort studies with lower diversities and abundances than matched bacteriomes; however, although fungi were detected in all examined cancer types, not all individual tumors were found positive for fungal signal (29). Others showed strong positive correlations between fungal and bacterial diversities, abundances, and co-occurrences in CRC and other cancers suggesting that tumor microenvironments (TMEs) are a permissive site for multi-domain microbial colonization (29, 35). Candida, Aspergillus, Fusarium, and Cryptococcus represent the leading genera in oral and oropharyngeal cancers (7, 36). Pancreatic tumors are associated with Malassezia (7, 37). In CRC, there were increases in Malassezia, Moniliophththora, Rhodotorula, Acremonium, Thielaviopsis, Pisolithus, *C. albicans,* and *C. tropicalis* and decreases in *S. cerevisiae* observed (7, 38). Candida was associated with the increased expression of pro-inflammatory immune pathways, particularly in stomach cancer (11). All these species belong to Dikarya Subkingdom, Ascomycota, and Basidiomycota phyla.

Accumulating studies have provided insight into the composition of the gut bacterial and fungal microbiota and their interaction and competition for nutrients, which might change metabolic processes associated with humans and laid the foundation for exploring how gut fungi are linked to—or even cause—diseases and how gut fungi can be manipulated to treat disease (28, 39). We aimed to conduct a systematic review to assess the role of fungal colonization in GI tumor formation focusing on key taxa, the ratio of fungal phyla Ascomycota to Basidiomycota, and alpha diversity, including human case–control studies evaluating inter- (healthy vs. cancer patient) and intraindividual (cancer tissue vs. normal adjacent tissue) differences.

## Materials and methods

We identified and assessed the included studies for a systematic review by the updated PRISMA 2020 statement (40).

### Search strategy

To obtain relevant studies, we scanned the Medline database by querying it using the PubMed interface. The final search was executed in March 2023. The basis of the query was the words “mycobiome,” “mycome,” “yeast,” “fungal,” and “mycobiot*” combined with the boolean “OR” operator. Filter categories for gut/intestinal/gastrointestinal, cancer, *progress, propagation, invasiveness* progression, and treatment options were applied with the “AND” operator to narrow the results. Terms not connected to the topic such as “microbiology” or “intestinalization” were removed from “All Fields” or “MeSH Terms” of the query translation. The final query was executed as a translation. The exact query and the translation details are provided in Supplementary material 1. The resulting query hits were saved in Pubmed format and imported into the abstract management software Rayyan (41). Studies qualifying for the full-text search were obtained through Semmelweis University subscription and stored in Rayyan. All Supplementary materials for the finally included (*n* = 14) studies were downloaded for data extraction.

### Study selection criteria

#### Inclusion criteria

Following the PICO framework, we pose the following question: In patients with GI cancer, is the presence of a malignancy associated with the diversity and composition of the mycobiome (especially the abundance of common taxa)? In line with the above, publications that met the following criteria were included:

(i) Patients were diagnosed with solid gastrointestinal (GI) cancers (either early or advanced stage), including gastric, CRC, pancreatic, HCC, or GI–neuroendocrine cancer. Patient participation in clinical trials is allowed.
(ii) Metagenomic (MG) sequencing or internal transcribed spacer (ITS) sequencing was performed using targeted PCR for fungal DNA from fecal, blood/plasma, or tissue samples.
(iii) An alpha diversity index and/or data on the phyla Ascomycota and/or Basidiomycota were published. In the absence of phylum-level data, prominent sub-taxas were also accepted.

#### Exclusion criteria

A publication was excluded if

(i) The publication type was review, systematic review, meta-analysis, editorial, correspondence, case series, case report, commentary, letter to editor, protocol, conference or abstract.
(i) The study is purely *in silico* or uses only database data.
(iii) The study concerns the topic of vaccination or vaccines.
(iv) It studies fungal infections in cancer patients due to immunosuppression (i.e., chemotherapy, neutropenia, neutropenia, medrol, and methotrexat).
(v) It describes skin-or lung-related fungal infections (dermatomycosis/pneumomycosis).
(vi)The study investigates the effect of fungi or their metabolites on cancer cells indirectly, or without the presence of the fungus in the gut (only studies of commensal, gut-colonizing fungi were included).
(vii) The study evaluates the anti-cancer effects of extracts of alimentary, non-microscopic fungi that are typically not part of the gut mycobiome.
(viii)The study involves digital pathology: histopathological, whole slide imaging (WSI) and machine learning analyses.

#### Selection process

Two authors (GSz and GySz) independently rated the abstracts based on the inclusion/exclusion criteria on a 3-point scale (include/exclude/maybe). The publication was transferred for a full-text review if the assessment resulted in a conflict. The authors assessed the publications with full information access in the full-text review. The remaining conflicts were resolved in the third stage of the screening process by the review supervisor (DD).

### Data extraction and risk of bias assessment

Two reviewers independently extracted data by carefully searching the full text, the Supplementary material, and external data archives/databases (GEO, ENA, SRA, git, and Zenodo) referenced. The descriptive information, first author, publication year, study type, compositional change in fungal taxa, number of cohorts and cohort size, sample type, cancer type, groups compared, cohort location, sequencing method, and data availability of the studies were extracted and summarized. The key parameters: Shannon index, A/B and F/B ratio, if available, were collected from the publication, and the Supplementary material, or calculated from raw data.

### Comparison of diversity measures

The primary outcomes in this study were interindividual (cancer vs. healthy control) or intraindividual differences (cancer vs. adjacent tissue, cancer vs. stool, and cancer vs. blood samples) in mycobiome alpha diversity (measured with the Shannon index) and composition [measured with the Ascomycota/Basidiomycota (A/B) ratio and/or changes in individual taxa, especially in Malassezia (A), Candida (A), Saccharomyces (A), and Aspergillus (A)].

Six studies published values of the Shannon index for their cohorts, and one more has published data from which we could calculate it. Values of the Shannon index were normalized, so all used a base 2 logarithm. For comparison, data from the healthy cohort of the Human Microbiome Project (4) and from the adenoma cohort of Luan et al. were gathered (42). In the case of the latter, since samples from adenoma and adjacent tissue did not differ significantly (Wilcoxon signed rank test *p* = 0.63), they were pooled. Furthermore, since the study used the UNITE database before its 2018 update, they could report data on 60 genus-level taxa with a high ratio of unclassified samples (54% on average). For better comparison with data analyzed based on newer, extended databases, the Shannon index was calculated from the more abundant operational taxonomic units (OTUs) (261) and not only from identified taxa, even though other included studies often used the latter.

Nine studies published compositional data in tabulated form or represented on bar plots and pie charts at the phylum level. The A/B ratios were estimated from figures extracted from the publication when available or calculated. The fungal composition of healthy cohorts in four studies was compared to reference healthy cohorts from 2013, 2017, and 2023 at the phylum level. The sign of a change in the A/B ratios from healthy/control state to cancer state was determined by prioritizing published numerical values and significance tests overestimation from figures.

### Quality assessment

The Newcastle–Ottawa scale (NOS) was used to assess numerically the methodological quality of the included observational studies (*n* = 14). Based on the Coding Manual for case–control and control studies, the aspects of selection and comparability of the study groups and exposure/outcome of interest were graded. None of the studies were scored with a high risk of bias (0–5) on NOS. The detailed scores given to each study are shown in Supplementary Table 3.

## Results

### Literature search and study characteristics

**Figure 1:**
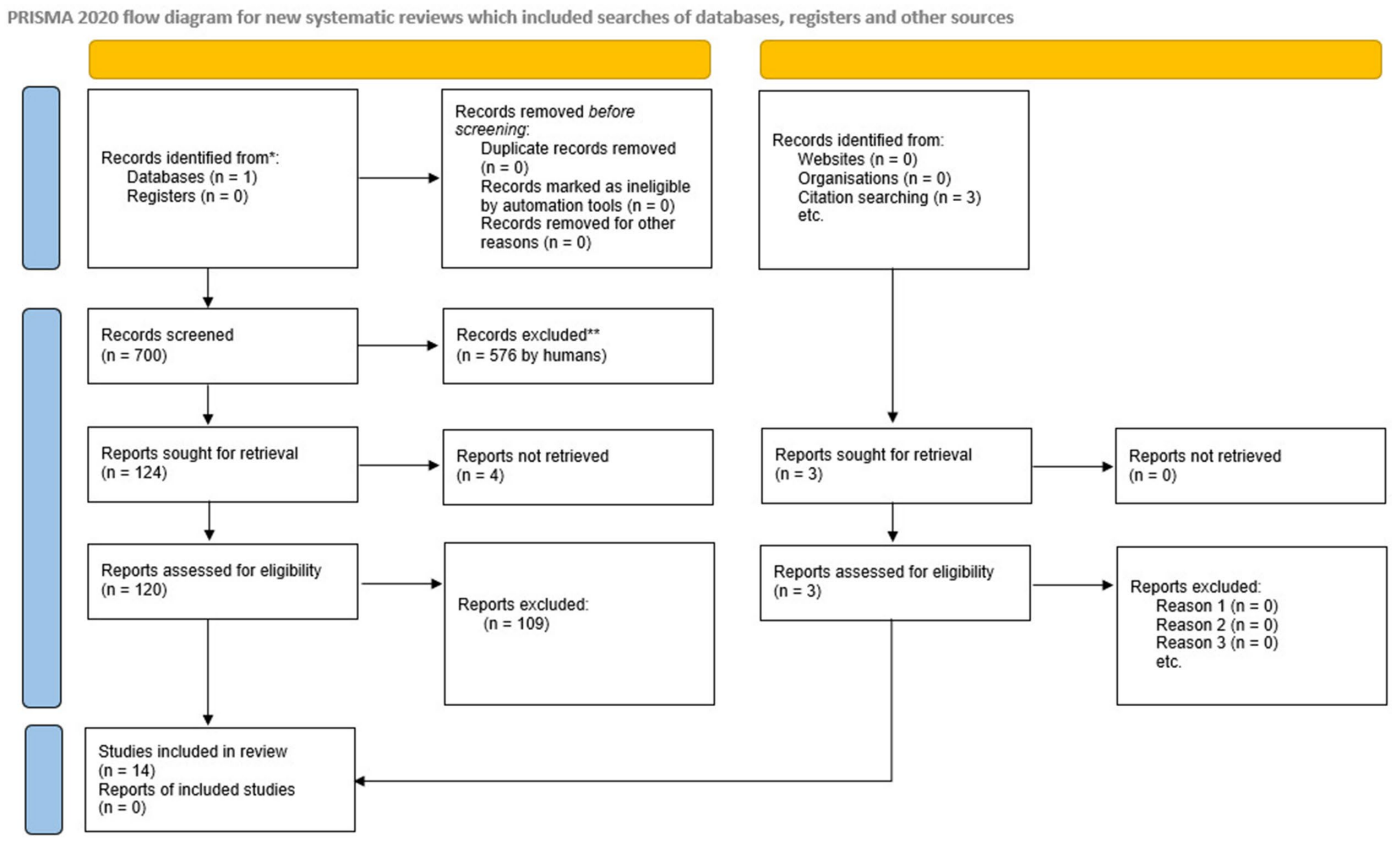
PRISMA flow chart of the search methodology for identifying relevant papers.

A total of 700 studies were identified by searching the Medline database using the Pubmed search engine. The detailed process of study selection is depicted in Figure 1. After screening the abstracts, 580 abstracts were excluded due to criteria mismatch. A total of 120 studies were retrieved for full-text screening and 109 were excluded due to a mismatch in criteria. Through the selection process, 11 studies fulfilled the criteria for systematic review; no studies were excluded due to ineligibility of outcome or study design. Three studies found from references while evaluating the systematically selected ones were also included and completed in the final 14 studies assessed in the review (11, 29, 37, 43–53).

### Descriptive characteristics of included studies

**Figure 2:**
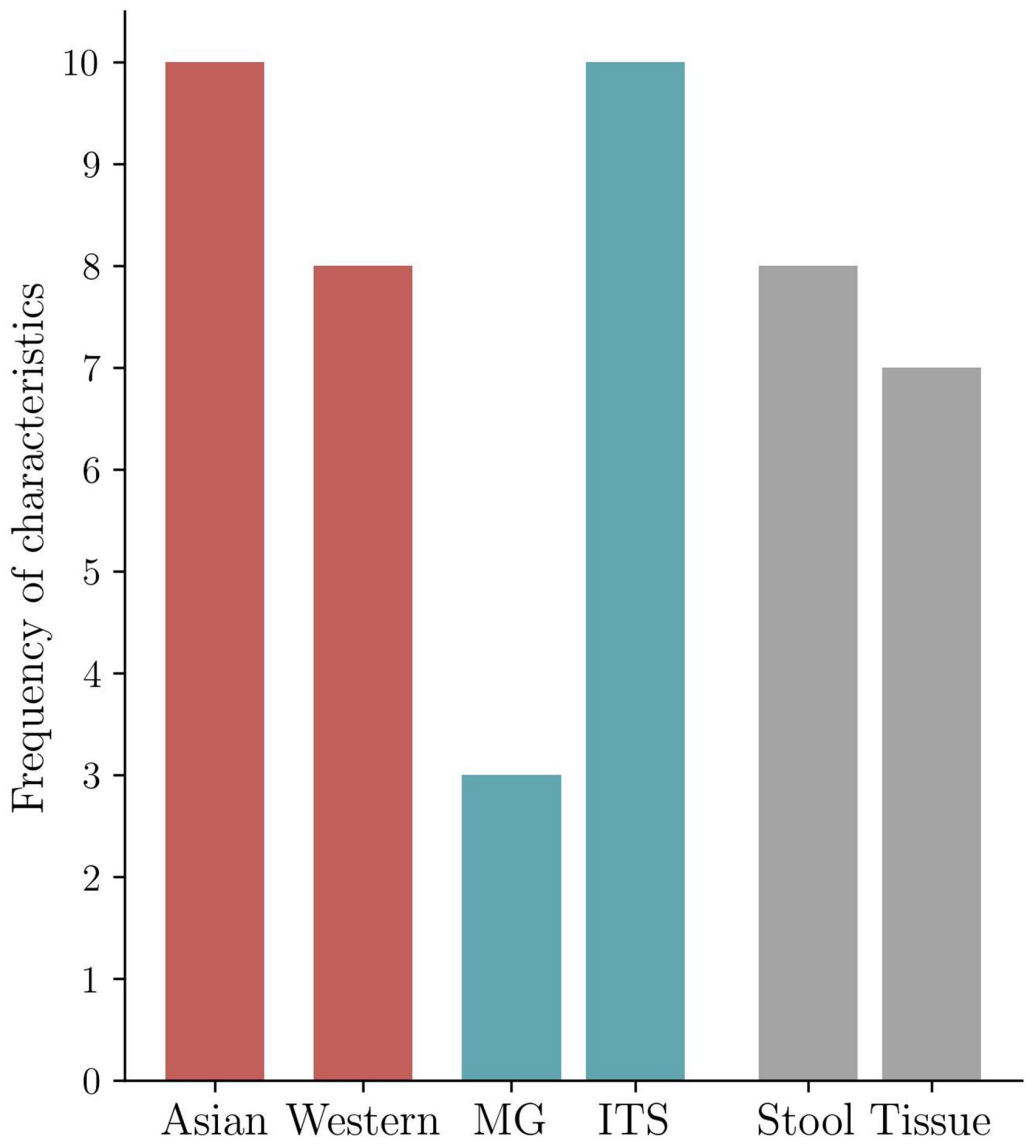
Frequency of the characteristics in the included studies. Geography-based distribution of included studies (*n* = 14) reported in the present investigation. Asia represents East Asian populations. Western includes *n* = 3 US mixed ethnical origin cohorts, *n* = 4 European cohorts with mainly Caucasian patients, and *n* = 1 cohort from the Near East. Distribution of the two most frequently applied molecular sequencing methods reported in the included studies (*n* = 14) (metagenomic (MG) vs. ITS). Distribution of type of specimen for detection of fungi in the included studies (*n* = 14) (tissue vs. stool). One of the included studies investigated multiple cohorts, the two primary sequencing methods are shown in the figure, and paired different type specimens are examined by one study; therefore, frequencies in the distribution will not add up to *n* = 14, the number of the included studies (Table 1; Supplementary Table 1).

The descriptive characteristics of the qualified 14 studies are summarized in Table 1. Samples included *n* = 8 stool and *n* = 7 cancer tissue samples that underwent sequencing analysis. Dohlman et al. also report the evaluation of blood samples, and Aykut et al. also use data from mouse models. The studies displayed three groups of sequencing approaches: *n* = 3 collected metagenomic data (MG), *n* = 10 internal transcribed spacer sequencing (ITS), and *n* = 1 18S sequencing data. According to the processed sequencing data, a taxonomy of mycobiome and bacteriome data is presented in *n* = 14 and *n* = 7 cases. For 8 of 14 studies, the raw sequencing data were available via the European Nucleotide Archive (ENA) or the Sequence Read Archive (SRA), and 3 studies kept the data under embargo at the time of the review process. The data of one study are only available upon request, and the data of two others do not comment on data availability. Asian (*n* = 10) and Western (*n* = 8) cohorts were investigated (Supplementary Table 1; Figure 2). Figure 2 shows the geography, major sequencing types, and sample types of included studies.

**Table 1:** Summary of general characteristics of included studies.

First Author	Year	Study type	Cancer	Sample type	Control	No. of patients	Sequencing	Primer-fwd	Primer-rev	Taxonomic assignment tools
Han	2022	Cohort study	CRC	Stool	Intraindividual (advanced adenoma)	52	MG			GATK PathSeq/NCBI (RefSeq, GenBank, Taxonomy)
N.-N. Liu	2022	Case control	CRC	Stool	Healthy	985	MG			Kraken 2/NCBI RefSeq, FungiDB, Ensemble
Li	2020	Case control	CRC	Stool	Healthy	281	18S			Uparse/Mothur
Gao	2017	Case control	CRC	Stool	Healthy	131	ITS2	ITS3	ITS4	Uparse/RDP(Ribosomal Database Project)
Yang	2022	Case control	GC	Cancer tissue	Healthy	65	ITS2	ITS3F-ITS4R	ITS2	Uparse/Unite
Zhang	2022	Cohort study	GC	Cancer tissue	Intraindividual (normal adjacent tissue)	61	ITS1-5F	ITS5-1737F	ITS2-2043R	Quiime 2/Unite
Zhong	2021	cohort study	GC	Cancer tissue	Intraindividual (normal adjacent tissue)	45	ITS2	ITS3_KYO2	ITS4	Uparse/RDP(ITS2)
Dohlman	2022	Cohort study	CRC	Cancer tissue	other (blood)	1759	ITS1	ITS1F	ITS2	GATK PathSeq/NCBI (RefSeq, GenBank, Taxonomy)
Aykut	2019	Cohort study	PDA		Intraindividual (gut of PDA patient)	13	ITS1	ITS1F	ITS2	Quiime, Vsearch/Unite
Z. Liu	2022	Cohort study	HCC		Intraindividual (cirrhosis)	28	ITS1	ITS1F	ITS2	Vsearch/Unite
Mohamed	2022	Case control	GEP_NEN		Healthy	34	ITS	ITS1	ITS4	Qiime/Greengenes, Unite
Coker	2018	Case control	CRC		Healthy	544	MG			Kraken (NCBI, FungiDB, Broad Institute, Ensembl)
Narunsky-Haziza	2022	Cohort study	CRC		Intraindividual (normal adjacent tissue)	1,183	ITS2	ITS86F	ITS4	Quiime 2/Unite, NCBI nr/nt databases
Richard	2018	Case control and cohort study	CRC		Intraindividual (normal adjacent tissue)	27	ITS2	ITS1F	ITS2	Uclust/Unite

CRC, colorectal cancer; GC, gastric cancer; PDA, pancreatic ductal adenocarcinoma; HCC, hepatocellular carcinoma; GEP-NEN, gastroenteropancreatic neuroendocrine neoplasm; ITS, internal transcribed spacer; MG, metagenome, Analysis pipeline/Databases for microbiome taxonomic assignment.

### Comparison of sequencing methodology and databases

Unclassified (unidentified) taxa were presented in 7 out of 14 of the included studies. Based on Table 1, the most frequently used database/software for the taxonomic assignment was UNITE (7 cases). Analysis pipelines using Kraken2 and the gatk suite (pathseq) for annotation were selected by 2 studies, each (54–56). Mothur, the Ribosomal Database Project, and ITS2 were also used to classify taxa in one study each (57–59). Sequences unidentified on the level of phylum were reported in *n* = 7 cases (11, 37, 44, 45, 50, 51, 53). Two studies have specified that they have dealt with unidentified sequences by utilizing the Basic Local Alignment Search Tool (BLAST) against [National Center for Biotechnology Information (NCBI)] databases and removing cases from taxonomic analysis if no match was found (29, 37). Both Dohlman et al. and Narunsky-Haziza et al. screened for contaminants and false-positive signals in their approach to retrieve microbial composition from The Cancer Genome Atlas (TCGA) cancer datasets (11, 29). Of the included studies, five used Uparse or Uclust algorithms for OTU clustering, which is included in the Usearch sequencing analysis pipeline. Other examples of pipelines used in the studies included Quiime, VSearch, Mothur, GATK, and PathSeq.

### Comparative analysis of results

At the phylum level, Ascomycota dominates the mycobiome in case–control and cohort studies, followed by Basidiomycota with a few exceptions. In the study of Richard et al., Basidiomycota was identified as the most abundant phylum. In the Japanese cohort in Liu et al., the second most abundant phylum was Mucoromycota (47). Analysis of sequencing data collected in TCGA for colon adenocarcinoma (COAD) and rectum adenocarcinoma (READ) also supports a higher abundance of Ascomycota than Basidiomycota throughout the large intestine (11).

Regarding lower taxonomic levels, studies highlight various taxa in association with GI cancers, but some trends can be observed. The genus Malassezia is reported to be elevated in relative abundance in many cancer types, with some results even hinting at a causal relationship between the presence of this genus and oncogenesis (37, 43, 44, 47, 51). These reports show considerable overlap with those that mention an increase in the genus Trichosporon (44, 46, 47, 51). In the gut, both of these are opportunistic pathogens, thus their elevated presence could be indicative of immunological deficiencies associated with cancer. Changes in the common genus Candida (esp. albicans) and Saccharomyces (esp. cerevisiae) are also often observed. Increased levels of Candida seem to be associated with GI cancers (11, 48, 49, 53). The relation of Saccharomyces to tumors is less clear. Han et al. report it in the top five abundant taxa in CRC, with others also detecting an increase (29, 45, 49). However, Li et al. observed Saccharomyces to be protective against disease progression of CRC, which is also supported by Dohlman et al. reporting an increase of the Candida/Saccharomyces ratio with progression into later stages (11, 46).

In addition to noting these compositional changes, multiple studies propose using fungi as biomarkers for GI cancers. Some highlight specific taxa for this purpose, namely Candida and Solicoccozyma (11, 52, 53). Others integrate fungal data into predictive algorithms (43, 47, 51). Of these, the studies of both Liu et al. and Coker et al. select *Talaromyces islandicus* and *Aspergillus rambellii* in their calculations for CRC. However, they report a different direction of change in the abundance of *T. islandicus* (down and up in cancer, respectively) (43, 47).

The Ascomycota/Basidiomycota (A/B) ratio was specifically studied by Gao et al., Coker et al. in CRC, and Yang et al. in GC. All three reported a significant difference in the A/B ratio in the cancer group compared to the healthy group (43, 44, 51). Based on these results, we assessed the A/B ratio in other studies as well, where it was accessible. The A/B ratio difference between cohorts was accessible numerically in the study of Gao et al. and Liu et al., directly from figures in Coker et al. and Yang et al., from raw data in the supplement of Narunsky-Haziza et al. and could be estimated from figures of Zhong et al., Zhang et al., Aykut et al. and Richard et al. (29, 37, 43, 44, 47, 50–53). We separated intra-and interindividual studies to cancel a putative bias of comparing case–control studies with cohort studies since significant differences were also observed in the A/B ratio between a possible transition state of cancer and a healthy state, e.g., in the study of Gao et al.

When assessing interindividual studies, we noticed that the A/B ratio in healthy controls was lower than in cancer patients in *n* = 2 cases, higher in *n* = 1 cases, and did not differ significantly for *n* = 2 cases. In the study of Yang et al., the A/B decrease is connected to thehigher rate of change of Basidiomycota compared to that of Ascomycota in the healthy to cancer group. In the study of Gao et al., the A/B increase is explained by the higher rate of Ascomycota growth compared to Basidiomycota in cancer and the healthy group.

**Figure 3:**
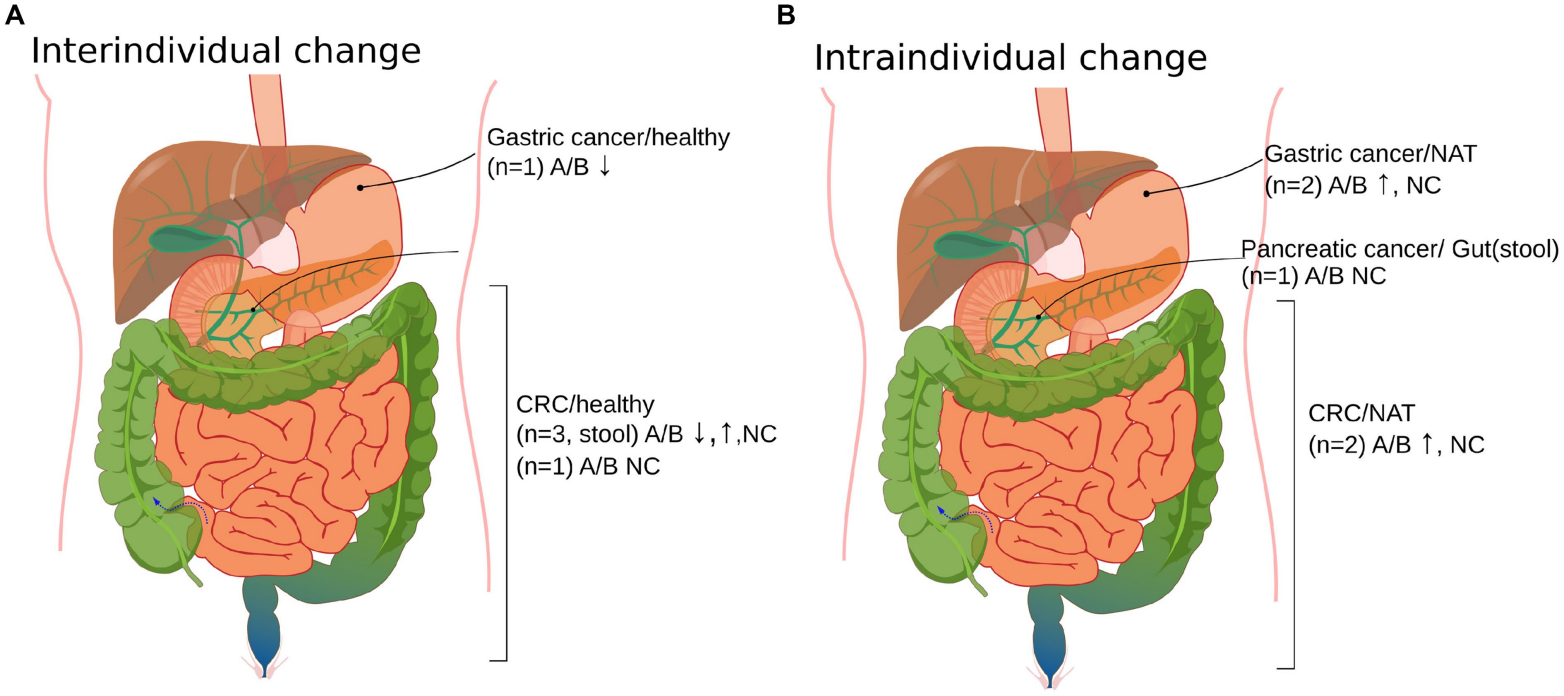
Organ site distribution of cancer vs. non-cancer controls of the Ascomycota/Basidiomycota (A/B) ratio in the included studies (*n* = 9). **(A)** Interindividual changes of mycobiota in cancer vs. healthy cases. **(B)** Intraindividual changes in cancer vs. control samples obtained from cancer patients. Normal adjacent tissue (NAT), colorectal cancer (CRC), no change (NC), and up and down arrows correspond to the increase and decrease of the A/B ratio from the non-cancer to cancer group. The sample used was cancer tissue in cases where it is not explicitly stated. The A/B ratios could be extracted from Aykut 2019 (37), Zhong 2021 (53), Zhang 2022 (52), Narunsky-Haziza 2022 (29), Richard 2018 (50), Gao 2017 (44), N.-N. Liu 2022 (47), Coker 2018 (43), and Yang 2022 (51). Supplementary Table 2 provides a tabulated version of Figure 3.

Regarding intraindividual studies, a higher ratio was shown in *n* = 2 cases and there was no difference in *n* = 2 cases in cancerous vs. normal tissue. The intraindividual comparison of tissue and stool A/B ratio in pancreatic cancer patients was also non-significant, as demonstrated by Aykut et al. (37) (Supplementary Table 2; Figure 3). The increase in intraindividual change is characterized by the growth of the Ascomycota abundance and the reduction of Basidiomycota abundance from normal to cancer tissue in studies of Zhong et al., while results of Narunsky-Haziza et al. show that the growth of both phyla contributes to A/B increase from normal to cancer state.

### F/B ratio

Studies by Liu et al. and Richard et al. investigated dysbiosis or interactions of the bacterial and fungal parts of the microbiome. Therefore, the Firmicutes/Bacteroidetes (F/B) ratio was also accessible in a healthy state to a colorectal cancer state (47, 50). The *n* = 5 cohorts in Liu’s study had a mean F/B ratio of 1.0 ± 0.5 (mean ± sd) in CRC and 2.0 ± 2.0 (mean ± sd) in healthy controls. The F/B and A/B ratios in the five cohorts showed moderate and weak correlations in the CRC groups (rho = 0.4) and the healthy controls (rho = −0.22), respectively. In the study of Richard et al., the increase of the F/B ratio from healthy to cancer state was larger in the case of sporadic CRC compared to colitis-associated CRC. Li et al. demonstrate that *Saccharomyces cerevisiae* feeding of antibiotics treated CRC model mice affects the bacteriome composition, an increase of the Firmicutes and Bacteriodetes relative abundance and the F/B ratio is caused compared to the control group that was fed with yeast extract peptone dextrose (46).

### Diversity of the mycobiome

**Figure 4:**
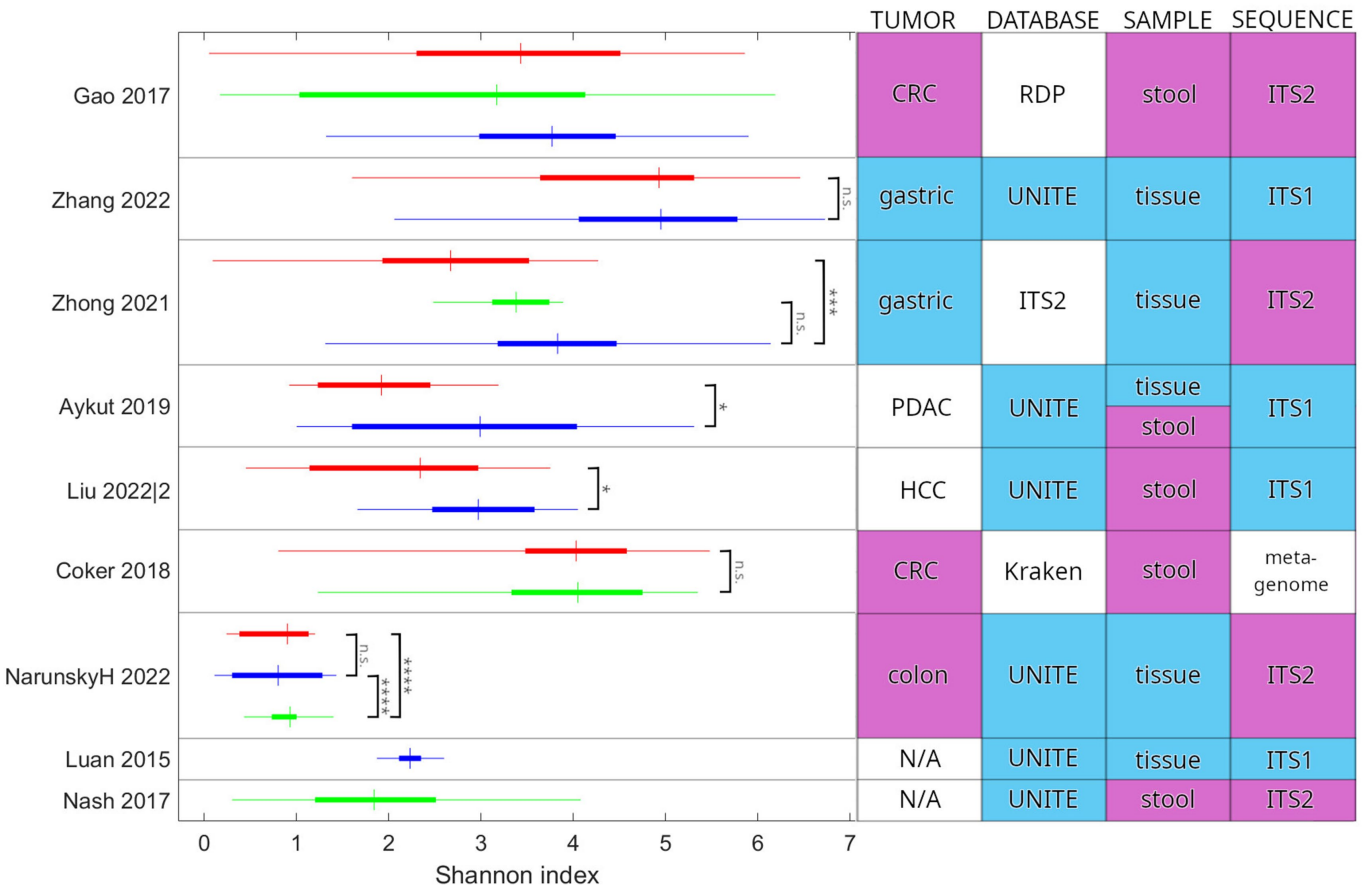
Comparison of the Shannon diversity index between studies. Study parameters that may influence the values and their comparison are also indicated. Colors in the table highlight the most common categories. Two additional studies are included for comparison: Luan et al. examined adenoma patients and Nash et al. reported on the healthy cohort of the Human Microbiome Project. Red indicates cancer-associated samples (tumor tissue or stool from the patient). Green indicates samples from healthy individuals. Blue indicates other samples, including normal adjacent tissue (NAT) and patients presenting with another chronic illness. In the horizontal bars, the thin line extends between the minimum and maximum, the wide portion is the IQR, and the crossbar is at the median value. Reported significance levels are indicated (n.s.: not significant, *p < 0.05, ***p < 0.001, ****p < <10^-6). Zhong et al. and Liu et al. have used an unpaired *t*-test. Aykut et al. and Coker et al. have used a Wilcoxon rank sum test. Gao et al. did not publish results for a statistical test of Shannon index values. Narunsky-Haziza et al. and Luan et al. did not publish values for the Shannon index. We calculated them from the published abundance values (species and OTU, respectively). We have used the Wilcoxon rank sum test to check statistical significance. N/A, not applicable; CRC, colorectal cancer; PDAC, pancreatic ductal adenocarcinoma; HCC, hepatocellular carcinoma; RDP, Ribosomal Database Project; ITS1, internal transcribed spacer 1; and ITS2, internal transcribed spacer 2.

The Shannon diversity index was the most commonly used diversity measure in the studies analyzed here. This index is affected by the number of categories (here: species or OTUs, a.k.a. richness) and the distribution of their relative abundance. The actual values reported show great variance, both within cohorts and between studies (Figure 4). There is a tendency for tumor-associated samples to present with lower diversity than other non-healthy samples from the same individual or patients with another chronic illness (statistically significant in three cases). We could not observe any association between study methodology and Shannon diversity. According to our results, inter-study consistency is modest in alpha diversity. However, a trend is present toward a decreased Shannon index in cancer vs. normal adjacent-or other healthy tissue. In addition, while tendencies in the population can be observed, both the complete range and interquartile range (IQR) of values are generally wide, calling into question the applicability of those tendencies on an individual level.

## Discussion

Although the mycobiome is approximately two orders of magnitude less abundant than the bacteriome, its impact on health and disease has been made undeniable by recent advances (39, 60). With constantly developing targeted amplicon sequencing and metagenomics, the diversity of colonizing and transient species and their role in homeostatic or imbalanced mechanisms is assessed more precisely (61). The interaction of the immunologically reactive part of the mycobiome with other microbiome components within and between sites is of emerging interest (62). We set the goal to investigate whether the routinely assessed mycobiome composition and diversity measures already have marker potential in GI cancer genesis or progression.

Types of major dysbiotic changes include the disappearance of healthy core species, increased abundance of pathogenic species, and decrease of diversity, which may occur simultaneously relative to the healthy homeostatic state (63). Nevertheless, the obvious dysbiosis observed in IBD raises the suspicion that in cancer evolution, there is also an intraindividual imbalance in the host compared to the physiological homeostasis. Ascomycetes have already been associated with IBD and IBS in this context (64, 65). Others showed an association between invasive yeast infections and diabetes, possibly through the dectin-1-dependent pathway because of the enrichment of *C. albicans* in newly diagnosed type 2 diabetes (T2D) (66). A significant increase of Candida was found in stool samples from patients with type 1 diabetes (T1D) and T2D compared to controls (67, 68). On the other hand, colonization by *C. albicans* appears to have several advantages for mammalian hosts: it protects against invasive fungal infections, stimulates the activation of innate immune cells, and protects against infection by antigenically unrelated bacterial pathogens (8). Altogether, colonizing fungi not only associate with difficult-to-treat infections and trigger allergic-type reactions but contribute to pathomechanisms of disease (8). Gut mycobiome dysbiosis without pathogenic colonization might be a risk factor in evaluating cancer formation in addition to the known risk factors.

The NIH Human Microbiome Project (HMP) characterized the healthy human subject (HHS) benchmark cohort also the taxonomy of the fungal microbiome of the GI tract (69). Nash et al. showed in their study that the gut mycobiome had lower alpha diversity (Shannon) than the bacteriome but with higher variability between patients in the healthy cohort of the HMP. No correlation between the bacteriome and mycobiome alpha diversity was detected. The healthy gut mycobiome at the phylum level is composed of approximately 89% Ascomycota and 10% Basidiomycota (4). The results of Maas et al. are consistent with those of Nash et al. (70). Both studies investigated fecal samples from cohorts with sample sizes of *n* = 146 and *n* = 163, respectively. Additionally, Maas et al. emphasize that the large variation in A/B ratio and relative abundance between samples is possibly caused by transient fungal species introduced through dietary habits with a remark that a follow-up study should be considered for clarification due to their single time point study design. Hoffmann et al. report that the presence of the phyla Ascomycota and Basidiomycota is exclusive of each other in healthy subjects, but this finding is not corroborated by other studies (71). Four of the studies included in our review had healthy cohorts with stool and tissue biopsy samples, where the relative abundance could be estimated and A/B compared to the reference cohort (44, 47, 50, 51). In the study of Yang et al., the A/B ratio is 8.3, similar to that of the HHS reference cohort. Yang et al. used non-cancer tissue biopsy which might result in the difference compared to stool samples of the healthy reference cohort. In contrast, colon tissues investigated by Richard et al. show a strong difference compared to the HHS cohort since here A/B < 1 because Basidiomycota’s relative abundance is larger than Ascomycota’s. Liu et al. and Gao et al. investigated stool samples of the healthy cohort and showed significant differences in the A/B ratio (4 and 21) compared with the HHS value which cannot be explained by taxonomic assignment since well-known databases across studies such as Kraken2, FungiDB, RDP, and NCBI Refseq distinct from UNITE were used with large difference in composition of unidentified phyla (Table 1).

Based on the systematic evaluation of published A/B ratios of GI cancers in intraindividual studies, it seems that the A/B ratio increases or exhibits no change from normal adjacent to tumor tissue in the case of GC and CRC. This might be important for exploring mycobial transfer between organs (e.g., stool vs. tissue or blood vs. tissue) (11, 37, 46). In the case of interindividual studies and CRC stool samples, slightly more studies supported an increase in the A/B ratio in cancer patients compared to healthy individuals (*n* = 2 vs. *n* = 1); however, the ratio is not decisive. Further studies on comparing paired stool, blood, tumor, and normal adjacent samples of cancer and healthy patients should clarify the possible application of the A/B ratio.

Dohlman et al. found that paired samples from tumors of the lower GI tract and blood had highly similar mycobial composition, possibly due to fungal DNA translocation from tumor tissue into blood circulation (11). Aykut et al. demonstrated hour-scale translocation of GFP-labeled *Saccharomyces cerevisiae* from gut to pancreas in a mouse model of pancreatic cancer. By comparing the fungal composition of human pancreatic ductal adenoma tissue and stool a difference was found at the diversity level. However, the relative abundance was non-significant for Ascomycota and Basidiomycota (37). In the former case, fungal translocation is promoted by the loss of tissue function caused by cancer growth, while in the latter one, it seems that fungal transport is part of the normal function. An example of a more thoroughly explored fungal–cancer interaction is the relation of Malassezia species with the immune system and its influence on tumor progression. Malassezia species may create pro-tumorigenic effects in pancreatic cancer, namely, through cell wall glycans inducing the activation of the complement system, affecting PDAC cells via membrane receptors (37). It may also affect the cytokine milieu, triggering IL-33 and IL-6 and promoting an immunosuppressive environment (72, 73).

In addition to the changes observed in fungal and bacterial taxa separately, there is evidence that the relationships between members of the two kingdoms are also different in cancer, with multiple studies reporting altered correlations (11, 43, 47). Liu et al. report increased interkingdom associations in CRC, e.g., that of *T. islandicus* of phylum Ascomycota with several bacterial species from phylum Bacillota (47). Dohlman et al. observe that GI tumors are dominated by either Candida or Saccharomyces species and that the presence of Candida spp. showed differential correlation with members of phylum Firmicutes (positive with the genus Dialister, and negative with genus Ruminococcus), and a negative correlation with species such as *Barnesiella intestinihominis* and *Akkermansia muciniphila*, which are generally considered to be beneficial to the host (11). The study of Coker et al. also supports a tendency for negative fungal–bacterial correlations in cancer compared to healthy controls. They describe a shift in the position of Proteobacteria, from positive correlation with Ascomycota to negative correlations with Basidiomycota and Mucoromycota. The relationship of the classes Eurotiomycetes, Leotimycetes, and Sodariomycetes to various bacteria also changes from positive to negative (43). The molecular mechanisms behind these differences are not yet explored.

Regarding the intra-study Shannon alpha diversity index differences, an interesting trend may be examined: we observed a tendency for cancerous samples to be lower in diversity than non-healthy samples included for comparison (three studies showed a significant difference and none in the other direction). One possible explanation is that the specifics of the tumor microenvironment are less permissive for fungal taxa than other environments, leading to lower abundance and thus the exclusion of taxa, based either on prevalence (lower abundance taxa have a higher chance to be excluded) or specific biological factors. Clear conclusions from the inter-study synthesis of the Shannon alpha diversity index could not be drawn. Whether the calculations were performed on identified species or OTUs (or some other grouping) is rarely stated that affects considerably the numerical value of the diversity index. Where both assigned species and OTUs were reported, we have found that diversity was higher in the case of the latter by approximately a factor of 2. In conclusion, direct inter-study comparison of the Shannon index faces limitations for currently available GI cancer studies.

The conclusions drawn from our systematic review warrant cautious interpretation. The methodology of the included studies differs greatly. Six studies used tissue samples from biopsies (11, 29, 37, 51–53), and eight collected stool samples (37, 43–49) (one study collected both). Mycobiome analysis was based on shotgun metagenomic sequencing in three studies (43, 45, 47), ITS1 sequence in four (11, 37, 48, 52), and ITS2 sequence in four (29, 44, 51, 53). One study analyzed the 18S sequence (46). All of these pose different challenges. Fungal species have a low relative abundance compared to bacterial ones (HMP reported 0.1–1%^4^), hindering detection by shotgun methods and leading to low sensitivity. Multiple attempts have been at developing computational techniques to alleviate this problem (74, 75). Intra-and interspecies differences in rRNA copy numbers may compromise the quantitative analysis of the mycobiome using ITS sequencing (76), and the choice of primers may introduce taxonomic biases due to mismatches (77). The variety of approaches is a confounding factor for any attempt at comparison or synthesis of results, including using open-access databases to pool sequencing data and perform a secondary cross-study analysis. This field of study would benefit greatly from standardization of methodology. Different comparisons of cancer/non-cancer or intra-and interindividual sites might serve as a starting point for the exploration of tumor progression markers.

## Conclusion

In the present study, we provide a comprehensive overview, including methodological and study design aspects of mycobiome studies in GI cancers. Mycobiota colonize cancer tissue which may play a crucial role in carcinogenesis. We believe this review provides a reasonable basis for future studies in the field. We conclude that the A/B ratio increases and mycobiome alpha diversity decreases in cancer compared to normal adjacent tissue. However, no significant differences were found regarding interindividual changes in the mycobiome. Genera Malassezia, Trichosporon, and Candida were the most associated taxa with GI cancers, while the role of Saccharomyces is controversial based on our systematic review.

## Data Availability

The original contributions presented in the study are included in the article/Supplementary material, further inquiries can be directed to the corresponding authors.
