# Supplementary material for: Predicting cancer-related mycobiome aspects in gastrointestinal cancers: a systematic review
Source: Front Med (Lausanne). 2024 Nov 29;11:1488377. doi: 10.3389/fmed.2024.1488377 (PMC11637848; doi:10.3389/fmed.2024.1488377)
Supplement: Supplementary file 1 [file Table_1.docx]

**Supplementary Table 1**. Cohort geography and data availability of the raw NGS data of the included studies.

| **First Author** | **Year** | **Geography** | **Raw NGS Data** |
| --- | --- | --- | --- |
| Han | 2022 | China | open access |
| N.-N. Liu | 2022 | 2 Asian, 3 Western | open access |
| Li | 2020 | China |  |
| Gao | 2017 | China | open access |
| Yang | 2022 | China | open access |
| Zhang | 2022 | China | embargo |
| Zhong | 2021 | China | embargo |
| Dohlman | 2022 | Western | open access |
| Aykut | 2019 | Western | open access |
| Z. Liu | 2022 | China | embargo |
| Mohamed | 2022 | Western | on request |
| Coker | 2018 | China |  |
| Narunsky-Haziza | 2022 | Western | open access |
| Richard | 2018 | Western | open access |

**Supplementary Table 2.** Summarization of A/B ratios extracted from the included studies where it was accessible. Direction of A/B change is from Control to Cancer group. Comparison type, cancer type and sample types of the groups are also shown. Abbreviations: GC: gastric cancer, PDA: pancreatic ductal adenocarcinoma, CRC: colorectal cancer, CG: Cancer Group, NAT: normal adjacent tissue.

| **First Author** | **Year** | **A/B change (Control ->Cancer)** | **Comparison** | **Cancer** | **CG Sample** | **Control Sample** |
| --- | --- | --- | --- | --- | --- | --- |
| **Aykut** | 2019 | NO CHANGE | intraindividual | PDA | tissue | stool |
| **Zhong** | 2021 | INCREASE | intraindividual | GC | tissue | NAT |
| **Zhang** | 2022 | NO CHANGE | intraindividual | GC | tissue | NAT |
| **Narunsky-Haziza** | 2022 | INCREASE | intraindividual | CRC | tissue | NAT |
| **Richard** | 2018 | NO CHANGE | intraindividual | CRC | tissue | NAT |
| **Richard** | 2018 | NO CHANGE | interindividual | CRC | tissue | tissue |
| **Gao** | 2017 | INCREASE | interindividual | CRC | stool | stool |
| **N.-N. Liu** | 2022 | NO CHANGE | interindividual | CRC | stool | stool |
| **Coker** | 2018 | DECRESE | interindividual | CRC | stool | stool |
| **Yang** | 2022 | DECRESE | interindividual | GC | tissue | tissue |

**Supplementary Table 3.** Numerical assessment of methodological quality of the included studies, based on the Newcastle-Ottawa scale for case-control/cohort studies. None of the studies show a high risk of bias (score of 0‑5).

| **FIRST AUTHOR** | **YEAR** | **SELECTION** | **COMPARIBILITY** | **EXPOSURE/ OUTCOME** | **SCORE** |
| --- | --- | --- | --- | --- | --- |
| Han | 2022 | ★★★★ | ★★ | ★★ | 8 |
| N.-N. Liu | 2022 | ★★★ | ★★ | ★★ | 7 |
| Li | 2020 | ★★★ | ★★ | ★★ | 7 |
| Gao | 2017 | ★★★★ | ★★ | ★★★ | 9 |
| Yang | 2022 | ★★★★ | ★★ | ★★★ | 9 |
| Zhang | 2022 | ★★★ | ★ | ★★★ | 7 |
| Zhong | 2021 | ★★★★ | ★★ | ★★ | 8 |
| Dohlman | 2022 | ★★★ | ★★ | ★★★ | 8 |
| Aykut | 2019 | ★★★ | ★★ | ★★ | 7 |
| Z. Liu | 2022 | ★★★ | ★★ | ★★ | 7 |
| Mohamed | 2022 | ★★★★ | ★★ | ★★★ | 9 |
| Coker | 2019 | ★★★ | ★★ | ★★★ | 8 |
| Narunsky-Haziza | 2022 | ★★★ | ★ | ★★★ | 7 |
| Richard | 2018 | ★★★★ | ★★ | ★★★ | 9 |
