# Supplementary material for: Predicting cancer-related mycobiome aspects in gastrointestinal cancers: a systematic review
Source: Front Med (Lausanne). 2024 Nov 29;11:1488377. doi: 10.3389/fmed.2024.1488377 (PMC11637848; doi:10.3389/fmed.2024.1488377)
Supplement: Supplementary file 2 [file Data_Sheet_1.DOCX]

**PubMed query and final search key used in the literature search.**

Mycobiome OR Mycome OR Yeast* OR Fungal OR Mycobiot* AND (Gut OR Intestinal OR Gastrointestinal) AND (Cancer OR Tumor* OR Tumour*) AND ((Progress* OR Aggressiveness OR Metast* OR Propagation OR Invasi*) OR (Radiotherap* OR Chemoradiotherapy OR Chemoradiation OR Chemotherap* OR Targeted therap* OR immunotherapy OR ICI OR immune checkpoint))

#(deleted from translation:"intestinalization"[All Fields],"intestinalized"[All Fields],"fungals"[All Fields],"microbiology"[MeSH Terms],"microbiology"[All Fields])

#Final search key:

("mycobiome"[MeSH Terms] OR "mycobiome"[All Fields] OR "mycobiomes"[All Fields] OR "Mycome"[All Fields] OR "yeast*"[All Fields] OR ("fungal"[All Fields] OR "fungi"[MeSH Terms] OR "fungi"[All Fields]) OR "mycobiot*"[All Fields]) AND ("gut"[Journal] OR "gut"[All Fields] OR ("intestinally"[All Fields] OR "intestinals"[All Fields] OR "intestine s"[All Fields] OR "intestines"[MeSH Terms] OR "intestines"[All Fields] OR "intestinal"[All Fields] OR "intestine"[All Fields]) OR ("gastrointestinal"[All Fields] OR "gastrointestinally"[All Fields] OR "gastrointestine"[All Fields])) AND ("cancer s"[All Fields] OR "cancerated"[All Fields] OR "canceration"[All Fields] OR "cancerization"[All Fields] OR "cancerized"[All Fields] OR "cancerous"[All Fields] OR "neoplasms"[MeSH Terms] OR "neoplasms"[All Fields] OR "cancer"[All Fields] OR "cancers"[All Fields] OR "tumor*"[All Fields] OR "tumour*"[All Fields]) AND ("progress*"[All Fields] OR ("aggress"[All Fields] OR "aggressed"[All Fields] OR "aggressing"[All Fields] OR "aggression"[MeSH Terms] OR "aggression"[All Fields] OR "aggressions"[All Fields] OR "aggressive"[All Fields] OR "aggressiveness"[All Fields] OR "aggressively"[All Fields] OR "aggressives"[All Fields] OR "aggressivity"[All Fields]) OR "metast*"[All Fields] OR ("propagable"[All Fields] OR "propagate"[All Fields] OR "propagated"[All Fields] OR "propagates"[All Fields] OR "propagating"[All Fields] OR "propagation"[All Fields] OR "propagations"[All Fields] OR "propagative"[All Fields] OR "propagator"[All Fields] OR "propagators"[All Fields]) OR "invasi*"[All Fields] OR ("radiotherap*"[All Fields] OR ("chemoradiotherapy"[MeSH Terms] OR "chemoradiotherapy"[All Fields] OR "chemoradiotherapies"[All Fields]) OR ("chemoradiotherapy"[MeSH Terms] OR "chemoradiotherapy"[All Fields] OR "chemoradiation"[All Fields]) OR "chemotherap*"[All Fields] OR (("target"[All Fields] OR "targetability"[All Fields] OR "targetable"[All Fields] OR "targeted"[All Fields] OR "targeting"[All Fields] OR "targetings"[All Fields] OR "targets"[All Fields] OR "targetted"[All Fields] OR "targetting"[All Fields]) AND "therap*"[All Fields]) OR ("immunotherapy"[MeSH Terms] OR "immunotherapy"[All Fields] OR "immunotherapies"[All Fields] OR "immunotherapy s"[All Fields]) OR "ICI"[All Fields] OR (("immune"[All Fields] OR "immuned"[All Fields] OR "immunes"[All Fields] OR "immunisation"[All Fields] OR "vaccination"[MeSH Terms] OR "vaccination"[All Fields] OR "immunization"[All Fields] OR "immunization"[MeSH Terms] OR "immunisations"[All Fields] OR "immunizations"[All Fields] OR "immunise"[All Fields] OR "immunised"[All Fields] OR "immuniser"[All Fields] OR "immunisers"[All Fields] OR "immunising"[All Fields] OR "immunities"[All Fields] OR "immunity"[MeSH Terms] OR "immunity"[All Fields] OR "immunization s"[All Fields] OR "immunize"[All Fields] OR "immunized"[All Fields] OR "immunizer"[All Fields] OR "immunizers"[All Fields] OR "immunizes"[All Fields] OR "immunizing"[All Fields]) AND ("cell cycle checkpoints"[MeSH Terms] OR ("cell"[All Fields] AND "cycle"[All Fields] AND "checkpoints"[All Fields]) OR "cell cycle checkpoints"[All Fields] OR "checkpoint"[All Fields] OR "checkpoints"[All Fields]))))
